# Brief Prescribing Support and Buprenorphine Adoption in Rural Primary Care: A Cluster Randomized Clinical Trial

**DOI:** 10.1001/jamanetworkopen.2026.3050

**Published:** 2026-03-25

**Authors:** Berkeley Franz, Lindsay Y. Dhanani, Daniel Brook, O. Trent Hall, Vivian Go, Janet E. Simon, Cheyenne Fenstemaker, William C. Miller

**Affiliations:** 1 Department of Social Medicine, Ohio University Heritage College of Osteopathic Medicine, Athens; 2 The Institute to Advance Population Health, Athens, Ohio; 3 School of Management and Labor Relations, Rutgers University, Piscataway, New Jersey; 4 Department of Psychiatry, University of Colorado Anschutz Medical Campus, Aurora; 5 Department of Psychiatry and Behavioral Health, Ohio State University Wexner Medical Center, Columbus; 6 Gillings School of Global Public Health, University of North Carolina at Chapel Hill, Chapel Hill; 7 Ohio University College of Health Sciences and Professions, Athens

## Abstract

**Importance:**

Although buprenorphine is highly effective at preventing overdose, access is limited in rural areas. Rural primary care is a critical site to expand access; prescribing support tailored for this setting is urgently needed.

**Objective:**

To test the feasibility and preliminary effectiveness of a brief buprenorphine prescribing support program (BPSP) tailored for rural primary care professionals (PCPs).

**Design, Setting, and Participants:**

This cluster randomized pilot clinical trial compared 27 Ohio community health centers with 63 PCPs (10 physicians, 50 nurse practitioners, and 3 physician assistants) receiving the support program at baseline and immediately after the intervention with those receiving only copies of the American Society of Addiction Medicine’s buprenorphine prescribing guidelines. Data were collected from July 25, 2024, to February 28, 2025.

**Interventions:**

The BPSP is a brief, hour-long, asynchronous and online training program, followed by an optional, live booster session, that provides clinical skills on buprenorphine prescribing, addresses misinformation on the safety of buprenorphine, and addresses stigma toward buprenorphine. Health centers were randomized with 2 centers allocated to the intervention for every 1 allocated to the control condition.

**Main Outcomes and Measures:**

Primary implementation outcomes were feasibility (measured via the 4-item Feasibility of Intervention Measure [scale of 1 to 5, with higher scores indicating greater feasibility]), acceptability (measured via the 4-item Acceptability of Intervention Measure [scale of 1 to 5, with lhigher scores indicating greater acceptability]), and appropriateness (measured via the 4-item Intervention Appropriateness Measure [scale of 1 to 5, with higher scores indicating greater appropriateness]) of the BPSP; willingness to treat opioid use disorder (OUD) in primary care (using a measure developed and tested with a different sample of PCPs); and likelihood of prescribing buprenorphine in the next 6 months (measured via a 5-point Likert scale ranging from 1.00 [extremely unlikely] to 5.00 [extremely likely]).

**Results:**

Of the 63 participating PCPs, 48 participants were allocated to the intervention condition and 15 to the control condition (49 female [78%]; mean [SD] age, 45.5 [11.4] years). Forty-nine participants (78%) practiced in rural areas and 10 (16%) had ever prescribed buprenorphine. Participants receiving the BPSP rated it as highly feasible (median score, 4.25 [IQR, 4.00-5.00]), acceptable (median score, 4.88 of 5.00 [IQR, 4.00-5.00]), and appropriate (median score, 5.00 of 5.00 [IQR, 4.00-5.00]). Participants receiving the BPSP had significantly higher willingness to treat OUD; 86% of rank comparisons improved post intervention. Intention to prescribe buprenorphine in the next 6 months also significantly increased; 98% of rank comparisons improved post intervention. Participants who completed the BPSP showed significant improvements in having correct information about buprenorphine, had greater confidence treating addiction and prescribing buprenorphine, and had lower stigma and greater empathy toward patients with OUD.

**Conclusions and Relevance:**

In this cluster randomized clinical trial of the BPSF, brief prescribing support was feasible, acceptable, and appropriate for implementation in rural primary care, and intentions to prescribe buprenorphine increased. A larger trial is needed to confirm preliminary findings.

**Trial Registration:**

ClinicalTrials.gov Identifier: NCT05505227

## Key Points

**Question:**

Is a brief buprenorphine prescribing support program designed for busy rural primary care offices feasible, acceptable, and effective at increasing willingness to treat opioid use disorder (OUD) and prescription of buprenorphine?

**Findings:**

In this cluster randomized clinical trial of 63 primary care clinicians in 27 rural community health centers, participants receiving the intervention rated the prescribing support program as highly feasible and acceptable. After the intervention, participants had higher willingness to treat OUD and to prescribe buprenorphine in the 6 months.

**Meaning:**

These findings suggest that tailored buprenorphine prescribing support for primary care may be a promising strategy to expand access to buprenorphine in rural areas.

## Teaser

This cluster randomized pilot clinical trial tests the feasibility, acceptability, and appropriateness of a buprenorphine prescribing support program for rural primary care professionals and assesses whether the program increases clinicians’ willingness to treat opioid use disorder and likelihood of prescribing buprenorphine.

## Introduction

Medications for opioid use disorder (OUD) profoundly reduce the risk of overdose^1,2^ and infectious disease transmission.^3^ Despite proven efficacy, fewer than 25% of people with OUD receive these medications.^4^ Access is particularly limited in rural areas; 33% of rural residents live in a county without buprenorphine access compared with 2% of urban residents.^5^ A promising solution is to engage rural primary care professionals (PCPs), including physicians, nurse practitioners (NPs) and physician assistants (PAs). The rural primary care setting provides a medical home, potentially reducing the stigma associated with OUD treatment.^6,7^

Expanding access to buprenorphine in rural areas requires addressing known barriers to prescribing, including longstanding regulation of the medication, inadequate clinical training, and stigma.^8,9,10^ There are additional barriers in rural areas, however, that must also be addressed, including training that has not been appropriately tailored for rural practice,^11^ limited peer mentorship opportunities,^12^ and a high chronic disease burden.^13^ The removal of the X-waiver requirement addressed a longstanding system-level barrier, yet buprenorphine prescribing has not meaningfully increased.^14^

Interventions at the clinician level that target stigma reduction and seek to increase knowledge hold promise for increasing access to buprenorphine. Like the X waiver, many existing interventions to increase buprenorphine prescribing have focused on clinical skills,^15^ which are necessary but not sufficient to improve buprenorphine adoption, as stigma may still prevent knowledgeable clinicians from prescribing.^16,17^ A social contact approach has been commonly used in stigma reduction interventions,^18,19^ but whether social contact improves willingness to prescribe buprenorphine remains unclear. Importantly, most stigma reduction efforts have focused on reducing stigma toward people with OUD. However, stigma related to buprenorphine prescribing is multifaceted and can be directed toward patients with OUD, the medications used to treat it, and/or those who prescribe it^9^; all must be addressed in tandem.

The goal of the present study was to design an intervention that paired clinical training on buprenorphine prescribing with multifaceted stigma reduction. We also sought to rigorously tailor the intervention for the busy rural primary care setting. We tested this program in a pilot cluster randomized clinical trial to assess implementation outcomes and determine preliminary effect sizes of changes in intentions to start prescribing to design a future determinative trial.

## Methods

### Study Setting and Randomization

The pilot cluster randomized clinical trial took place in 27 community health centers, either federally qualified health centers (FQHCs) or “look-alikes,” which meet the same criteria as FQHCs but do not receive special funding, within 6 health center systems in Ohio. We recruited community health centers with the help of the Ohio Association of Community Health Centers. Health centers received the intervention consisting of the buprenorphine prescribing support program (BPSP) or copies of the American Society of Addiction Medicine (ASAM) buprenorphine prescribing guidelines in the control condition.^20^ Health centers were sequentially numbered and randomized using simple randomization with 2 health systems allocated to the intervention for every 1 control health system. Randomization was conducted by the study biostatistician (J.E.S.), and neither the study team nor participants were blinded. Clinics were enrolled by the principal investigator (B.F.) in collaboration with Ohio Association of Community Health Centers, and clinic leaders facilitated individual participant enrollment through an email invitation. Inclusion criteria for participation were being a physician, NP, or PA working in primary care at a clinic participating in the trial. Data were collected via an embedded survey immediately preceding and after completion of the initial training module. Control condition clinics completed baseline surveys at the same time as intervention clinics. Implementation trial data were collected between July 25, 2024, and February 28, 2025.

This study followed the Consolidated Standards of Reporting Trials (CONSORT) reporting guideline for reporting randomized clinical trials. The study protocol was reviewed and approved by the Ohio University Institutional Review Board, and all participants provided consent electronically through an embedded survey question. The trial protocol is provided in Supplement 1. A data safety and monitoring board oversaw the study. No adverse events were reported.

### Intervention

#### Overview

The BPSP is a brief, scalable implementation strategy to support buprenorphine adoption in rural primary care. We collaborated with rural PCPs, community advocates, and health systems leaders to codevelop the BPSP. We conducted 2 statewide surveys in Ohio and in-depth interviews with rural PCPs and clinic leaders to develop the BPSP. We then pilot-tested it with 23 PCPs and completed another round of in-depth interviews afterward to assess needed changes to the program. This step resulted in the inclusion of a buprenorphine take-home guide for prescribers. We then finalized the program with a community advisory board.

**Figure 1.**
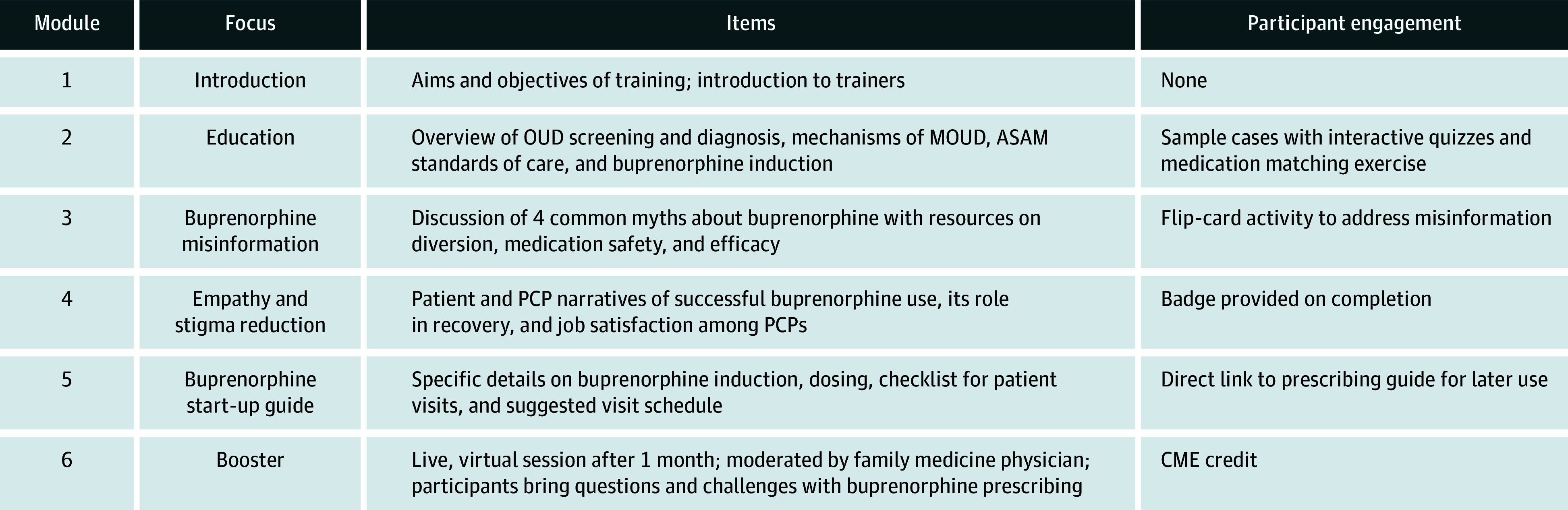
Overview of the Buprenorphine Prescribing Support Program ASAM indicates American Society of Addiction Medicine; CME, continuing medical education; MOUD, medicines for opioid use disorder; OUD, opioid use disorder; PCP, primary care professional.

Building on existing buprenorphine prescribing interventions that emphasize clinical skills, training in the BPSP addresses OUD screening, OUD diagnosis, and induction and maintenance of buprenorphine treatment. Novel elements of the BPSP include a section on addressing common myths about buprenorphine, and comprehensive stigma reduction using a social contact approach featuring peer rural buprenorphine prescribers and people who have received the medication (Figure 1). The BPSP was also rigorously tailored for the rural setting, integrating specific forms of stigma reduction unique to rural PCPs^9^ and addressing rural-specific barriers to buprenorphine prescribing.^11^ In line with ASAM guidelines,^20^ the BPSP uses a low-threshold approach to buprenorphine prescribing, which aims to reduce barriers for medication access and provide treatment in convenient locations.^21,22^

#### Conceptual Framework

The BPSP built on 2 empirically established methods to reduce stigma. We drew from intergroup contact theory,^23^ which posits that people hold more favorable attitudes toward members of their own group and that facilitating contact with members of an outgroup can improve attitudes.^24^ We also drew from cognitive dissonance theory,^25^ which suggests that people are motivated to change their beliefs and behaviors if they receive new information that challenges existing beliefs so that attitudes and behaviors become more aligned over time. These theories have been used successfully to change workplace attitudes and behavior^25,26^; they informed the development of the BPSP.

#### Structure

The BPSP is a 1-hour asynchronous training method delivered on the learning management platform Articulate 360. Clinic leaders sent participants a web link to access the training, and participants had 30 days to complete it. Approximately 30 days after completing the BPSP, a single, optional 1-hour live, virtual booster was offered using a group format. This module was facilitated by an addiction medicine–trained family physician and offered the opportunity to address ongoing prescribing questions.

#### Control

Participants in the control condition received an electronic link to the ASAM Buprenorphine Prescribing Guidelines^20^ and no further implementation support. Participants in the intervention condition did not receive these guidelines, although the guidelines served as a foundation for the clinical portion of the BPSP.

### Data Sources

#### Primary Implementation Outcomes

Primary implementation outcomes were feasibility, acceptability, and appropriateness. A score of 4.00 of 5.00 was considered sufficient to proceed to a future confirmatory trial. Feasibility, the ease with which a new intervention can be used, was measured using the 4-item Feasibility of Intervention Measure (scale of 1 to 5, with higher scores indicating greater feasibility).^27^ Acceptability, the extent to which an intervention is agreeable or satisfactory, was assessed using the 4-item Acceptability of Intervention Measure (scale of 1 to 5, with higher scores indicating greater acceptability).^27^ Appropriateness, the perceived alignment of the intervention within a particular practice setting, was measured with the 4-item Intervention Appropriateness Measure (scale of 1 to 5, with higher scores indicating greater appropriateness).^27^ We also measured intentions to begin buprenorphine prescribing. For PCPs who had never prescribed, we measured the likelihood of prescribing in the next 6 months using a 5-point Likert scale ranging from 1.00 (extremely unlikely) to 5.00 (extremely likely). For PCPs who had previously prescribed, we measured the likelihood of prescribing to additional patients in the next 6 months using the same Likert scale. We also measured general willingness to treat OUD in primary care using a measure developed and tested with a different sample of PCPs.^28^

#### Secondary Outcomes

We measured knowledge of the mechanisms of buprenorphine, naltrexone, and methadone using 3 previously developed and tested multiple-choice board-style questions. These were coded dichotomously as 2.00 for correct and 1.00 for incorrect.^29^ We measured misinformation using an expanded version of the endorsement of Buprenorphine Misinformation Scale, which was previously validated with a different sample of PCPs.^30^ This scale measures endorsement of correct information with 9 items using a 5-point Likert scale ranging from 1.00 (strongly disagree) to 5.00 (strongly agree), with 5.00 indicating more correct information. Confidence treating substance use was measured using 4 items on different forms of OUD screening and management using a 5-point Likert scale ranging from 1.00 (strongly disagree) to 5.00 (strongly agree), with 5.00 indicating greater confidence. Confidence prescribing buprenorphine was similarly measured with 2 items on induction of buprenorphine therapy and continuing buprenorphine prescribing for patients already on the medication. These measures were adapted from previous studies on the screening, brief intervention, and referral to treatment model and self-efficacy among health care practitioners.^31,32^

We measured attitudes toward people with OUD, including empathy, using a 6-item scale developed by Batson and colleagues.^33^ We previously adapted and validated this scale to measure empathy toward people with OUD among a separate sample of PCPs.^18^ This scale contains a series of feelings that represent empathy (eg, sympathetic, compassionate, warm) and asks participants to indicate the extent to which they feel each of these feelings toward people with OUD. Responses are provided on a scale from 1.00 (not at all) to 7.00 (extremely), with 7.00 indicating greater empathy. We measured stigma toward people with OUD using a scale that was adapted from another scale assessing bias toward people who inject drugs.^34,35^ The scale contains 10 items that reflect explicit negative attitudes toward people with OUD (eg, “People with opioid use disorder are immoral”). Responses were provided on a scale of 1.00 (strongly disagree) to 5.00 (strongly agree), with 5.00 indicating high stigma. eTable 1 in Supplement 2 lists all study measures.

Demographic indicators, including race and ethnicity, were self-reported by participants. Race and ethnicity were collected to ensure equitable representation in research and in compliance with funding guidelines and were categorized as Black, Hispanic of any race, White, and multiracial. Rurality was defined using the Ohio Department of Health’s county designation.^36^ Fully rural and partially rural counties were coded as rural.

### Statistical Analysis

We used descriptive statistics to characterize participant demographic characteristics and outcomes at each time point. To assess baseline differences between control and intervention conditions in the trial dataset, Kruskal-Wallis tests were used. Given the small sample sizes and nonnormal outcome distributions, Wilcoxon signed rank tests were used to examine within-participant changes among participants in intervention clinics before and after receiving the BPSP. Because this was a pilot trial with small, sparse clusters (2-3 participants), analyses were conducted at the individual participant level and did not adjust standard errors for clustering by clinic; therefore, the results should be interpreted as exploratory. The threshold for statistical significance was *P* < .05 using 2-tailed tests. Statistical significance refers to within-group change before and after intervention among participants in intervention clinics unless otherwise specified. Eight participants were missing after the intervention and were dropped using listwise deletion. Missing participants were slightly more likely to be nonprescribers, but all other demographic characteristics were similar to those of the other participants. All analyses were conducted using Stata, version 19 (StataCorp LLC).

## Results

**Figure 2.**
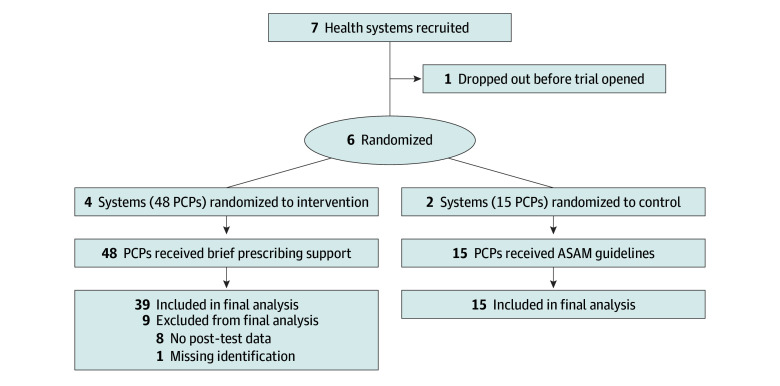
Study Flow Diagram ASAM indicates American Society of Addiction Medicine; PCP, primary care professional.

Before the trial, a total of 23 participants piloted the BPSP (eTables 2 and 3 in Supplement 2). In the pilot cluster randomized clinical trial, we enrolled 27 community health centers across 6 health center systems (eTable 4 in Supplement 2). Sixty-three participants completed the trial, with 15 in the control condition and 48 in the intervention condition (Figure 2). There were no significant differences at baseline between participants in either condition. Trial participants were predominantly rural (49 [77.8%]). Fifty participants were NPs (79.4%), 10 (15.9%) were physicians, and 3 (4.8%) were PAs. Few had ever prescribed buprenorphine (10 [15.9%]) (Table 1). All trial participants were employed at FQHCs; 49 participants (77.8%) were female, 13 (20.6%) were male, and 1 (1.6%) was nonbinary (mean [SD] age, 45.5 [11.4] years). One participant (1.6%) was Asian, 2 (3.2%) were Black, 2 (3.2%) were Hispanic of any race, 59 (93.7%) were White, and 1 (1.6%) identified as multiracial. Forty-eight participants (mean [SD] age, 43.5 [11.1] years) were allocated to the intervention condition and 15 to the control condition (mean [SD] age, 51.7 [10.3] years).

**Table 1.** Participant Demographic Characteristics

Characteristic	Pilot trial group, No. (%)	
	Control (n = 15)	Intervention (n = 48)
Age, mean (SD), y	51.7 (10.3)	43.5 (11.1)
Sex		
Female	13 (86.7)	36 (75.0)
Male	2 (13.3)	11 (22.9)
Nonbinary	0	1 (2.1)
Race and ethnicity		
Asian	0	1 (2.1)
Black	0	2 (4.2)
Hispanic	1 (6.7)	1 (2.1)
White	15 (100)	44 (91.7)
Multiracial	0	1 (2.1)
PCP type		
Physician	3 (20.0)	7 (14.6)
Nurse practitioner	12 (80.0)	38 (79.2)
Physician assistant	0	3 (6.3)
Employed at FQHC	15 (100)	48 (100)
Rural practice	12 (80.0)	37 (77.1)
Previously prescribed buprenorphine	1 (6.7)	9 (18.9)

Abbreviations: FQHC, federally qualified health center; PCP, primary care professional.

### Primary Outcomes

Trial participants rated the BPSP as highly feasible (median score, 4.25 [IQR, 4.00-5.00]), acceptable (median score, 4.88 [IQR, 4.00-5.00]), and appropriate (median score, 5.00 [IQR, 4.00-5.00]) (Table 2). Participants receiving the BPSP had significantly higher willingness to treat OUD after receiving the BPSP (median score, 4.13 [IQR, 3.00-5.00] at baseline vs 4.00 [IQR, 3.25-5.00] after the intervention; *P* = .04); 86% of the rank comparisons post intervention were higher than preintervention scores. Intention to prescribe buprenorphine in the next 6 months significantly increased from a median score of 2.00 (IQR, 1.00-3.00) to 2.00 (IQR, 1.00-4.00; *P* = .006) after the intervention; 98% of postintervention scores ranked higher than their corresponding preintervention scores.

**Table 2.** Changes in Outcomes After the BPSP

Outcome	Pilot trial group		
	Control (n = 15)	Intervention	
		Baseline (n = 48)	After BPSP (n = 39)
Primary implementation score, median (IQR)^a^			
Feasibility	NA	NA	4.25 (4.00-5.00)
Acceptability	NA	NA	4.88 (4.00-5.00)
Appropriateness	NA	NA	5.00 (4.00-5.00)
Willingness to treat OUD, median (IQR)^b^	4.00 (3.00-4.75)	4.13 (3.00-5.00)	4.00 (3.25-5.00)^c^
Likely to begin prescribing, median (IQR)^b^	2.00 (2.00-4.00)	2.00 (1.00-3.00)	2.00 (1.00-4.00)^d^
Knowledge of mechanisms, No. (%)			
Buprenorphine knowledge	8 (53.3)	32 (66.7)	31 (79.5)
Methadone knowledge	9 (60.0)	27 (56.3)	29 (74.4)
Naltrexone knowledge	7 (46.7)	32 (66.7)	28 (71.8)
Correct information, median (IQR)^e^	3.56 (3.11-4.44)	3.67 (3.11-4.00)	4.33 (3.78-4.67)^d^
Confidence prescribing buprenorphine, median (IQR)^f^	2.50 (1.00-3.00)	2.25 (1.00-5.00)	3.50 (2.50-5.00)^d^
Confidence diagnosing and treating SUD, median (IQR)^f^	4.25 (3.50-4.75)	4.25 (3.75-5.00)	4.75 (4.00-5.00)^b^
Stigma, median (IQR)^g^	2.10 (1.90-2.80)	2.20 (1.70-2.65)	2.10 (1.70-2.40)^d^
Empathy, median (IQR)^h^	3.50 (3.00-3.67)	3.42 (2.92-4.08)	4.00 (3.33-4.50)^d^

Abbreviations: BPSP, buprenorphine prescribing support program; OUD, opioid use disorder; SUD, substance use disorder.

^a^ A score of 4.00 of 5.00 was considered sufficient to proceed to a future confirmatory trial.

^b^ Scores range from 1.00 to 5.00, with higher scores indicating higher likelihood.

^c^ *P* <.05.

^d^ *P* <.001.

^e^ Scores range from 1.00 to 5.00, with 5.00 indicating more correct information.

^f^ Scores range from 1.00 to 5.00, with 5.00 indicating greater confidence.

^g^ Scores range from 1.00 to 5.00, with 5.00 indicating higher stigma.

^h^ Scores range from 1.00 to 7.00, with 7.00 indicating greater empathy.

### Secondary Outcomes

Trial participants showed modest improvements in knowledge of the mechanisms of medications for OUD, although not all changes were statistically significant. Thirty-two of 48 participants who received the BPSP (66.7%) could correctly identify the mechanism of buprenorphine at baseline compared with 31 of 39 (79.5%) after the intervention (*P* = .11). Twenty-seven of 48 participants (56.3%) could identify the mechanism of methadone at baseline compared with 29 of 39 (74.4%) after the BPSP (*P* = .03). Thirty-two of 48 PCPs (66.7%) could identify the mechanism of naltrexone at baseline compared with 28 of 39 (71.8%) after the BPSP (*P* = .44). Trial participants endorsed significantly more correct information about buprenorphine after the intervention (median score, 4.33 [IQR, 3.78-4.67]) than at baseline (median score, 3.67 [IQR, 3.11-4.00]; *P* < .001).

**Figure 3.**
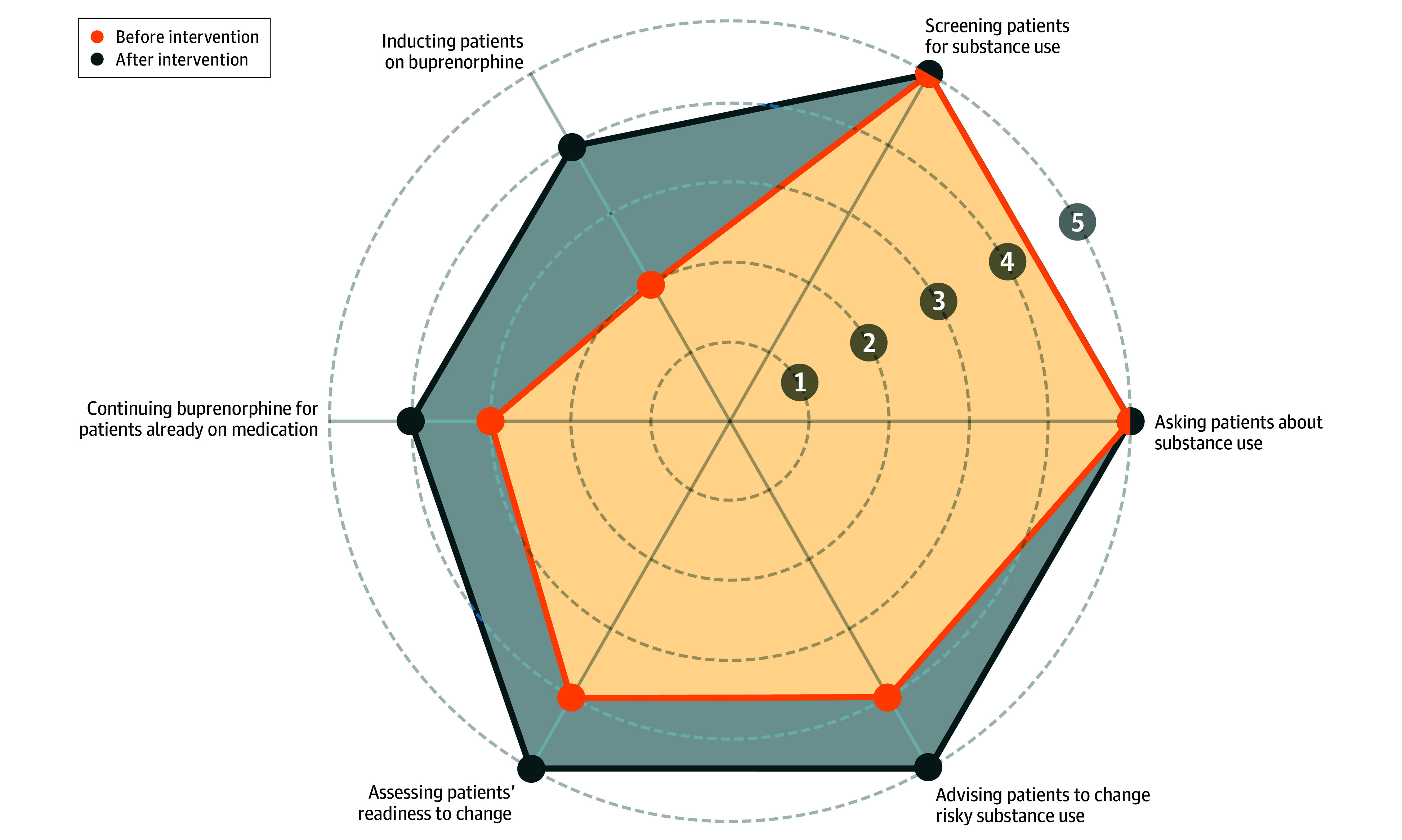
Network Graph Showing Changes in Confidence Before and After the Intervention Minimum is 1; maximum, 5.

Trial participants had greater confidence treating substance use disorders after the intervention (median score, 4.75 [IQR, 4.00-5.00]) compared with baseline (median score, 4.25 [IQR, 3.75-5.00]; *P* < .001). Similarly, PCPs had greater confidence prescribing buprenorphine after the intervention (median score, 3.50 [IQR, 2.50-5.00]) compared with baseline (median score, 2.25 [IQR, 1.00-5.00]; *P* < .001). The largest improvements in confidence were for prescribing buprenorphine (Figure 3).

Trial participants held less stigma toward people with OUD after receiving the intervention (median score, 2.10 [IQR, 1.70-2.40]) compared with baseline (median score, 2.20 [IQR, 1.70-2.65]; *P* = .005). Trial participants also had greater empathy toward people with OUD after the intervention (median score, 4.00 [IQR, 3.33-4.50]) compared with baseline (median score, 3.42 [IQR, 2.92-4.08]; *P* < .001).

## Discussion

Participants who completed the BPSP rated the brief training program as highly feasible, acceptable, and appropriate for rural primary care. There are limitations to analyzing pilot data for effectiveness,^37^ but we aimed to assess preliminary effect sizes of changes in OUD treatment willingness as well as detect signals that the BPSP may improve buprenorphine implementation.

We found evidence that participants endorsed less misinformation after completing the BPSP. Participants who completed the BPSP also expressed increased confidence in screening and assessing patients for OUD and prescribing buprenorphine. Participants who completed the BPSP also had improved attitudes toward people with OUD, including significantly greater empathy toward this patient population and less stigma. Most importantly, participants who completed the BPSP were significantly more willing to provide care for people with OUD in primary care and, among those who did not currently prescribe buprenorphine, their intention to start prescribing in 6 months significantly increased.

That attitudes improved after a brief intervention is encouraging, given the challenges with reducing stigma and fostering empathy.^38^ Stigma toward people with OUD is a well-documented barrier to implementing buprenorphine within primary care.^9,39,40,41^ The BPSP uses a social contact approach, an evidence-based strategy for reducing stigma,^18^ that was tailored for rural PCPs. Specifically, we exposed rural PCPs to patients who had experienced long-term remission from opioid use with buprenorphine, as well as other rural PCPs who have prescribed buprenorphine. These findings suggest that using social contact approaches to improve comfort with stigmatized groups, such as patients with OUD and buprenorphine prescribers, may be an important strategy to improve attitudes and potentially increase buprenorphine adoption.

In addition to stigma toward people with OUD, buprenorphine itself is stigmatized, limiting prescribing.^9^ To address this form of stigma, we used an educational approach in the BPSP to reduce misinformation about the safety and appropriateness of buprenorphine for treating OUD. Improving knowledge about the mechanisms of buprenorphine may be associated with greater willingness to prescribe the medication.^29^ Our study findings suggest that brief educational interventions aimed at improving knowledge and reducing misinformation about stigmatized medications should be further tested as a possible implementation strategy.

The BPSP also aims to improve clinical skills related to screening and assessing the severity of OUD, as well as completing buprenorphine induction. Limited training among the health professions on treating addiction and prescribing medications for OUD is a recognized barrier to buprenorphine prescribing.^8,11,42,43^ Confidence treating addiction in rural primary care was high at baseline but also improved somewhat after completing the BPSP. Confidence for buprenorphine prescribing showed greater improvements, although it remained relatively low after the BPSP. Additional prescribing support will likely be necessary for a subset of rural PCPs. Peer prescriber mentorship models that allow for ad hoc consultations as well as prescribing advice^12,15^ may be an adjunctive strategy to improve prescribing confidence and help sustain improvements in confidence over time.

The BPSP was developed as an implementation strategy focused on changing clinician-level willingness to prescribe buprenorphine. Beyond individual clinicians, additional prescribing barriers remain at the organizational and structural levels.^44,45^ Organizational-level implementation strategies such as practice facilitation^46^ may also be necessary, but these may be taxing for small primary care clinics. Continued policy advocacy to address structural barriers to buprenorphine access is critical.^47^

### Strengths and Limitations

A strength of this study is the inclusion of intentions for future prescribing in addition to knowledge and attitudes. Trials with longer follow-up are needed to show translation of intent into prescribing behavior and to better understand whether the booster session or other supports are necessary to sustain improved attitudes and knowledge over time.

This pilot study also has some limitations, including its small sample size. Although we enrolled the planned 27 community health centers, these clinics were very small, with some only having 2 to 3 PCPs on staff; a larger confirmatory trial is needed. By design, we developed the BPSP for rural PCPs and tested it primarily with this group, given profound disparities in access to buprenorphine in rural areas. Fourteen participants in this trial (22.2%) were not from rural clinics, however. Future studies should not only confirm whether the BPSP is effective in rural settings but also should explore whether it needs to be tailored for diverse practice locations. The BPSP also needs to be tested and possibly adapted for other types of health care professionals, including practitioners in obstetrics and gynecology, pediatrics, and other specialties where buprenorphine implementation has been limited.

## Conclusions

In this cluster randomized clinical trial, we developed a clinician-focused prescribing support program that improves on existing programs as it also addresses misinformation about buprenorphine and uses a social contact approach to reduce stigma toward the medication, prescribers, and patients who receive it. The BPSP was also rigorously tailored for rural primary care, where implementation barriers are unique. Our pilot trial findings suggest that the BPSP is feasible, acceptable, and appropriate for rural primary care and may improve attitudes toward buprenorphine and willingness to prescribe. If it improves prescribing behavior in a larger trial, the BPSP has the potential to improve buprenorphine access in critically important rural areas.

## Notes

Supplement 1.
Trial Protocol and Statistical Analysis Plan

Supplement 2. **eTable 1.** Study Measures **eTable 2.** Pilot Study Participant Demographics **eTable 3.** Changes in Outcomes After Initial Buprenorphine Prescribing Support Program Pilot Testing **eTable 4.** Characteristics of the Trial Clinics

Supplement 3.
Data Sharing Statement
