# Supplementary material for: Brief Prescribing Support and Buprenorphine Adoption in Rural Primary Care: A Cluster Randomized Clinical Trial
Source: JAMA Netw Open. 2026 Mar 25;9(3):e263050. doi: 10.1001/jamanetworkopen.2026.3050 (PMC13019231; doi:10.1001/jamanetworkopen.2026.3050)
Supplement: Supplement 1. — Trial Protocol and Statistical Analysis Plan [file jamanetwopen-e263050-s001.pdf]

Section 4: Protocol Synopsis

4.1 Study Design

4.1.a. Detailed description

Rural counties, particularly in Appalachia, are battling fast-growing outbreaks of HIV. One of the most promising mechanisms for reducing HIV transmission in these communities is the use of buprenorphine which treats opioid use disorder and also decreases HIV risk. Despite the potential benefit of buprenorphine, health care professionals (HCPs) in rural areas of the United States are much less likely to have received the training and federal waiver necessary to prescribe this medication. The single biggest obstacle to extensive MOUD use is stigma—stigma among communities, families of people who use drugs (PWUD), and remarkably, among HCPs.

We recently completed a study demonstrating that OUD-related stigma is common among physicians in Ohio. This stigma is greater among physicians practicing in rural areas—the same areas experiencing the greatest impact of the opioid crisis. Stigma is also associated with fewer buprenorphine prescribers, syringe service programs, and naloxone dispensaries in the surrounding county. Physicians' stigma toward PWUD is predicted by 3 key attitudes that are unique to the opioid crisis and which require tailoring of existing training interventions.

Rural health care professionals would benefit from brief stigma-reduction training interventions but to date effective stigma-reduction interventions have not been tailored specifically for MOUD or for the rural primary care setting. Adapting MOUD-specific interventions that are feasible and acceptable for health care professionals practicing in rural areas is critical for improving access to buprenorphine in underserved rural communities.

This study includes developmental activities aimed at isolating the modifiable features of OUD-related stigma among rural health care professionals, adapting a brief stigma-reduction training intervention and finalizing the intervention with health care professional feedback, and implementing a two-arm randomized pilot trial among rural health care professionals to assess feasibility and acceptability of the intervention. We hypothesize that the brief stigma-reduction training intervention will be both feasible and acceptable in the rural primary care setting. Our specific aims are to:

Aim 1: Examine HCP knowledge and attitudes about OUD to understand their reluctance to prescribe MOUD and manage patients with OUD.

Aim 2: Develop a narrative-based stigma reduction intervention and tailor it to the rural primary care setting using HCP feedback.

Aim 3: Assess the feasibility and acceptability of a stigma-reduction intervention in a pilot study in a diverse group of rural primary care HCPs.

To adapt existing interventions, we will disseminate a focused survey to 300 rural primary care physicians in Ohio, assessing key features of stigma and the relationship to buprenorphine attitudes and use. Prior to screening, we will also pre-test all survey items with 25 health care professionals with similar characteristics to our target population. The pilot will assess survey length, and check response distribution, item comprehension, and data collection procedures.

To assess intervention components, we will provide the intervention to 15 rural health care professionals and conduct a structured focus group to explore each module, assess perceptions of the intervention, identify the most salient features, and isolate barriers of concern to inform the development of a booster module.

To understand perceptions of each component in greater depth, we will conduct in-depth interviews with 15 rural health care professionals who have received the intervention.

Following completion of the booster module, we will provide the intervention to 15 rural additional health care professionals and conduct a structured focus group to assess perceptions of the booster module, identify needed changes to each module in the overall intervention, and finalize the intervention for feasibility testing.

Screening, enrollment, randomization and assessments. We will enroll 400 HCPs across 6 rural FQHCs who (1) currently practice in Ohio and (2) are eligible buprenorphine prescribers under federal law (3) do not currently prescribe buprenorphine at full capacity. Primary care providers will include physicians with board certification in internal or family medicine as well as family nurse practitioners. Because our primary outcomes are intervention feasibility, clinics will be cluster randomized into a group of 4 (intervention arm, n=267) and 2 (control arm, n=133) to increase the amount of pilot data on the intervention. We will recruit practices from Federally Qualified Health Centers (FQHCs) and FQHC look-alikes in rural, Ohio counties affiliated with OACHC. All eligible participants in enrolled practices will be screened for eligibility and asked to complete the study.

Enrolled participants will be evaluated at baseline, immediately following the intervention, and at 3 and 6 months after enrollment. The baseline and follow-up questionnaires will be administered via an online survey.

Follow-up in-depth interviews will be conducted to monitor the process of change in primary and secondary outcomes over time. Over the enrollment period, we will select 10 participants in the intervention arm. We will purposively select 4 individuals who newly prescribed buprenorphine during this period, 3 individuals who were not retained in the study, and 3 individuals who did not prescribe buprenorphine at all. Interviews will be conducted by key personnel who are trained interviewers. Each interview will last approximately 60 minutes.

4.1.b. Primary purpose — Health Services Research

4.1.c. Interventions

Name: Brief OUD Stigma-Reduction Training Intervention

Description: The Brief OUD Stigma-Reduction Training Intervention is comprised of several modules aimed at decreasing stigma, increasing knowledge about medications for OUD, and increasing willingness to prescribe buprenorphine among rural HCPs currently in practice. The first module provides an introduction to the aims of training and the study overview. Module 2 provides education about OUD in the rural context, current standards of care, and buprenorphine prescribing and regulation. Module 3 includes a narrative-based interview with a patient in recovery from OUD, focusing on the context of opioid initiation and the role that buprenorphine played in recovery. Module 3 also includes a second narrative-based interview with a primary care provider who was initially hesitant to work with this patient population and prescribe buprenorphine but currently does so and finds the work meaningful. The final module provides resources for additional training and a direct link to X waiver certification as well as a reminder about future data collection points. Participants will complete interactive quizzes after each module and continuing education or continuing medical education (CE or CME) credit will be provided after completion of the full training. An interactive, booster module will be provided after one month which focuses on anticipated problems and barriers faced by providers prescribing buprenorphine.

Study Phase: N/A

Interventional Study Model: Parallel

Masking: Yes, Investigator, Outcomes Assessor

Allocation: Randomized

Outcome Measures:

Type	Name	Time Frame	Brief Description
Primary	Acceptability	3 months	Acceptability is the perception that the intervention is agreeable, palatable, or satisfactory to health care professional participants. Acceptability will be assessed qualitatively and quantitatively.<br><br>We will administer to participants the Acceptability of Intervention Measure (AIM), which comprises 4 items containing responses on a 5-point Likert scale. We will also record participants' perceptions and feedback about the intervention modules. In in-depth interviews, we will explore the stakeholders' perceptions of the content of the intervention, the need for it in rural practice settings, its potential effectiveness, and ease of delivery
Primary	Feasibility	3 months	Feasibility refers to the ease in which a new intervention can be used within a specific setting. It will be measured using the 4-item FIM. We will also measure feasibility through interviews with health care professional participants to understand barriers and facilitators of implementing the intervention in the rural primary care setting.
	Appropriateness	3 months	Appropriateness refers to the perceived alignment of the intervention within a particular practice setting and will be measured by the 4-item Intervention Appropriateness Measure (IAM). We will also measure appropriateness in in-depth interviews with health care professionals, exploring the most salient features of the intervention and its fit with current training needs.
Primary	Adoption	6 months	Adoption refers to intentions or actions to adopt an EBI. We will measure adoption as whether a HCP initiates buprenorphine prescribing for the first time or increases their patient panel (yes/no) and as the total number of patients receiving buprenorphine for each HCP. This measure will also allow us to measure preliminary effect sizes related to buprenorphine prescribing which are critical for the

			follow-up cluster randomized trial to test intervention effectiveness.
Secondary	Training and prescribing waivers	6 months	Whether the participant completed any additional training (yes/no) and whether the participant received an X-waiver (yes/no)
Secondary	Prescribing knowledge	6 months	Knowledge of buprenorphine standards of care
Secondary	Willingness to work with patients with OUD	6 months	5-item scale (α=0.87) assessing willingness to work with patients with OUD.
Secondary	Attitudes toward patients with OUD	6 months	10-item scale (α=0.81) assessing bias toward patients with OUD, adapted from a previously validated scale measuring bias toward people who inject drugs.

Subject participation duration: 24 months

Power Analyses

4.3 STATISTICAL DESIGN AND POWER

Number of subjects we expect to enroll for the quantitative assessments:

400 provider participants

Expected effect size and power:

Given that this is a pilot study that will be used to power a future R01, we do not have sufficient data about the psychometric properties of the outcome measures utilized in this study among our population of interest, including the means and standard deviations. As such, we cannot estimate the specific degree of change we can detect in terms of scale points. Thus, the sample size estimation is based on a differences-in-differences cluster randomized trial with two parallel arms and a continuous outcome variable. Alpha was set to 0.05 and power was estimated at 80%. We estimate retention to be 90% at 6 months, due to limited job turnover within community health centers enrolled in the study. Based on these assumptions, a sample of 400 provides 80% power to detect a difference of d = 1.01 with participants clustered in 6 clinics. This indicates we will have sufficient power to assess a difference of one standard deviation in each of the primary outcome variables.

Statistical Methods

Quantitative data analyses

All tests will be based on a nominal 5% two-sided type I error probability. Confidence intervals will have nominal 95% coverage. All primary analyses will compare arms by measures of feasibility.

Initial Data Inspection:
Data will be analyzed to evaluate assumptions to inform the most valid statistical analysis. Data distributions will be assessed so that appropriate transformations may be applied where indicated and to check for outliers. Data will be examined for completeness to identify any possible systematic bias with respect to group and in particular, attrition. Analyses will be conducted using SAS v9.4. Analysis details for each hypothesis are given below.

Analyses of primary outcomes:
Buprenorphine prescribing outcomes.

We will apply a generalized linear mixed model (GLMM). The GLMM will include subject ID as a random effect and clinic as a fixed effect; the appropriate link function will be used, invoking generalized estimating equations (GEE) to account for the longitudinal (repeated measures) aspect of the data. Using GEE accounts for the repeated-measures design and uses all available data, including participants who may have missed time points and accounts for the correlation between repeated outcome measures at two time points (at 3 and 6 months). We will specify a Gaussian error distribution and identity link to compare the means of each feasibility outcome between the two arms. The primary model will estimate effects across all time points; a secondary model will include an interaction term with time to estimate effects at 3 and 6 months separately.

Analyses of secondary outcomes:
Analysis of secondary outcomes (willingness to treat and harm reduction knowledge and attitudes) between the two arms will follow the same analysis approach as above.

Missing data:
Strategies to minimize loss to follow-up and missing data will be undertaken in partnership with the Ohio Association of Community Health Centers who are partnering to recruit and retain participants in the study.

For any outcome, if we are missing >10% of observations, we will assess predictors of loss to follow-up and apply multiple imputation under the assumption that data are missing at random (MAR) conditional on observed data. When disseminating results, we will present both complete-case and imputed results along with a description of the accompanying assumptions.
