# Supplementary material for: Brief Prescribing Support and Buprenorphine Adoption in Rural Primary Care: A Cluster Randomized Clinical Trial
Source: JAMA Netw Open. 2026 Mar 25;9(3):e263050. doi: 10.1001/jamanetworkopen.2026.3050 (PMC13019231; doi:10.1001/jamanetworkopen.2026.3050)
Supplement: Supplement 2. — eTable 1. Study Measures eTable 2. Pilot Study Participant Demographics eTable 3. Changes in Outcomes After Initial Buprenorphine Prescribing Support Program Pilot Testing eTable 4. Characteristics of the Trial Clinics [file jamanetwopen-e263050-s002.pdf]

Supplementary Online Content

Franz B, Dhanani LY, Brook D, et al. Brief prescribing support and buprenorphine adoption in rural primary care: a cluster randomized clinical trial. *JAMA Netw Open.* 2026;9(3):e263050. doi:10.1001/jamanetworkopen.2026.3050

eTable 1. Study Measures

eTable 2. Pilot Study Participant Demographics

eTable 3. Changes in Outcomes After Initial Buprenorphine Prescribing Support Program Pilot Testing

eTable 4. Characteristics of the Trial Clinics

This supplementary material has been provided by the authors to give readers additional information about their work.

© 2026 Franz B et al. *JAMA Network Open*.

eTable 1. Study Measures

Outcome	Instrument	# Items	Response Scale	Source
Willingness to treat OUD	Willingness scale	5	1-5 Likert	Franz, Dhanani, and Brook, 2021. Adapted from therapeutic attitudes scale by Cartwright, 1980.
Likely to begin prescribing	Likelihood to begin prescribing measure	1	1-5 Likert	Developed for this study
Likely to increase prescribing	Likelihood to increase prescribing measure	1	1-5 Likert	Developed for this study
Feasibility	Feasibility of Intervention Measure	4	1-5 Likert	Weiner et al., 2017
Acceptability	Acceptability of Intervention Measure	4	1-5 Likert	Weiner et al., 2017
Appropriateness	Intervention Appropriateness Measure	4	1-5 Likert	Weiner et al., 2017
Buprenorphine Knowledge	Board-style question	1	Correct/Incorrect (Scored 2/1)	Piscalko et al., 2024
Methadone Knowledge	Board-style question	1	Correct/Incorrect (Scored 2/1)	Piscalko et al., 2024
Naltrexone Knowledge	Board-style question	1	Correct/Incorrect (Scored 2/1)	Piscalko et al., 2024
Correct information	Endorsement of buprenorphine misinformation scale (expanded)	9	1-5 Likert	Franz et al., 2024

© 2026 Franz B et al. *JAMA Network Open*.

Confidence prescribing buprenorphine	Confidence prescribing buprenorphine measure	2	1-5 Likert	Developed for this study. Adapted from Elwy, Horton, and Saitz, 2013 and Harris and Yu, 2016.
Confidence diagnosing and treating SUD	Confidence diagnosing and treating SUD measure	4	1-5 Likert	Developed for this study. Adapted from Elwy, Horton, and Saitz, 2013 and Harris and Yu, 2016.
Stigma	Stigma scale	10	1-5 Likert	Franz, Dhanani, and Miller, 2021. Adapted from Brener and von Hippel, 2008.
Empathy	Empathy scale	6	1-7 Likert	Dhanani et al., 2023. Adapted from Batson's Empathy Adjectives by Batson et al., 1988.

© 2026 Franz B et al. *JAMA Network Open*.

eTable 2. Pilot Study Participant Demographics

	**Pre/Post Test No. (%) (n=94)**
Previously prescribed buprenorphine	27 (28%)
Physician	24 (26%)
Nurse Practitioner	65 (69%)
Physician Associate	5 (5%)
Female	70 (74%)
Employed at FQHC	85 (90%)
Asian	2 (2%)
Black	5 (5%)
Hispanic	3 (4%)
White	85 (90%)
More than one race	2 (2%)
Rural practice	63 (67%)

Notes: FQHC=Federally qualified health center

© 2026 Franz B et al. *JAMA Network Open*.

eTable 3. Changes in Outcomes After Initial Buprenorphine Prescribing Support Program Pilot Testing

**Outcomes**	**Pre-Post Dataset Median (IQR)**	
	**Pre-test n=94**	**Post-test n=64**
Willingness to treat OUD	4.00 (3.20-4.20)	4.20 (3.80-5.00)**
Likely to begin prescribing	2.00 (1.00-3.00)	3.00 (2.00-4.00)***
Likely to increase prescribing	5.00 (4.00-5.00) (n=27	5.00 (5.00-5.00) (n=21)
Feasibility	n/a	4.88 (4.00-5.00)
Acceptability	n/a	5.00 (4.25-5.00)
Appropriateness	n/a	5.00 (4.00-5.00)
Buprenorphine Knowledge	66%	83%*
Methadone Knowledge	66%	81%*
Naltrexone Knowledge	74%	76%
Correct Information	4.00 (3.38-4.63)	4.88 (4.13-5.00)***
Confidence prescribing buprenorphine	2.50 (1.00-4.50)	4.00 (3.00-5.00)***

© 2026 Franz B et al. *JAMA Network Open*.

Confidence diagnosing and treating SUD	4.5 (3.75-5.00)	5.00 (4.25-5.00)***
Stigma	2.30 (2.00-2.70)	2.10 (1.80-2.30)***
Empathy	3.50 (2.83-4.00)	4.00 (3.17-4.50)***

Note: OUD= opioid use disorder; SUD= substance use disorder; IQR= inter-quartile range; Significance levels are indicated by *=<.05, **<.01, ***<.001

© 2026 Franz B et al. *JAMA Network Open*.

eTable 4. Characteristics of the Trial Clinics*

Health system	Study arm	No. of clinics		Rural clinics, No. (%)	No. of participants
		Total system	Enrolled in trial		
1	Intervention	17	7	7 (100)	23
2	Intervention	8	2	2 (100)	5
3	Intervention	10	4	0	11
6	Intervention	8	6	6 (100)	9
4	Control	7	1	0	3
5	Control	16	7	7 (100)	12

*Includes 27 trial clinics.

© 2026 Franz B et al. *JAMA Network Open*.
