# Supplementary material for: Brief Prescribing Support and Buprenorphine Adoption in Rural Primary Care: A Cluster Randomized Clinical Trial
Source: JAMA Netw Open. 2026 Mar 25;9(3):e263050. doi: 10.1001/jamanetworkopen.2026.3050 (PMC13019231; doi:10.1001/jamanetworkopen.2026.3050)
Supplement: Supplement 3. — Data Sharing Statement [file jamanetwopen-e263050-s003.pdf]

Data Sharing Statement

Franz. Brief Prescribing Support and Buprenorphine Adoption in Rural Primary Care. *JAMA Netw Open*. Published March 25, 2026. doi:10.1001/jamanetworkopen.2026.3050

Data
Additional Information: NCT05505227
Data available: Yes
Data types: Deidentified participant data, Data dictionary
How to access data: De-identified data will be made available upon reasonable request to franzb@ohio.edu
When available: With publication

Supporting Documents
Document types: Informed consent form
How to access documents: Informed consent information will be made available upon reasonable request to franzb@ohio.edu
When available: With publication

Additional Information
Who can access the data: Data will be made available to researchers whose proposed use of the data has been approved.
Types of analyses: Data will be made available for any research purposes.
Mechanisms of data availability: Data will be made available with a signed data use agreement.
